# Coaches’ perspectives on training quality in elite para-sport: a qualitative study of coach–athlete collaboration

**DOI:** 10.1186/s13102-026-01788-5

**Published:** 2026-06-10

**Authors:** Vemund Øvstehage, Jan Kocbach, Cecilia Severin, Knut Skovereng, Silvana Bucher Sandbakk, Øyvind Sandbakk

**Affiliations:** 1 Centre for Elite Sports Research, Department of Neuromedicine and Movement Science, Norwegian University of Science and Technology, Trondheim, Norway; 2 Department of Sport Science and Physical Education, University of Agder, Kristiansand, Norway; 3 School of Sport Science, UiT The Artic University of Norway, Tromsø, Norway; 4 Department of Physical Performance, Norwegian School of Sport Sciences, Oslo, Norway

**Keywords:** Para-athletes, Disability, Impairment, Training process, Paralympics, Sport performance

## Abstract

**Background:**

Training quality is essential for performance development among elite para-athletes. Despite coaches’ central role in structuring and adapting training, little research has explored their perspectives on what constitutes high-quality training. This study investigates which factors elite para-sport coaches perceive as important for achieving high training quality in daily practice.

**Methods:**

Eight Norwegian coaches with experience at the Paralympic and World Championship level participated in semi-structured interviews. A reflexive thematic analysis was conducted using a hermeneutic-phenomenological approach to identify shared meanings across coaches’ lived experiences.

**Results:**

Four overarching themes emerged as central to training quality: (1) a supportive training environment (including adapted facilities and integrated support teams), (2) organization and adaptation of the training process (emphasizing collaborative planning and load management), (3) coaches’ understanding of the athlete and sport (highlighting impairment-specific and sport competence), and (4) athlete ownership of training (fostering autonomy and responsibility). These factors were interrelated and influenced both the long-term training process and individual sessions. Coaches described training quality as a dynamic outcome of collaboration between coach, athlete, and support staff, involving continuous dialogue, real-time adjustments, and shared decision-making.

**Conclusion:**

Elite para-sport coaches perceive training quality as a multifaceted and collaborative process. Key contributions include a well-adapted environment, flexible planning, individualized understanding of athlete needs, and athlete engagement. These findings offer practical insights for enhancing coaching practices in para-sport and underscore the importance of coach–athlete–support team synergy in optimizing performance and well-being.

**Supplementary Information:**

The online version contains supplementary material available at 10.1186/s13102-026-01788-5.

## Background

Training quality can be defined as the degree of excellence related to how athletes execute the training process or training sessions [1]. Accordingly, high training quality may optimize training adaptations, improve performance and prepare the athlete to reach competition goals. Two interconnected dimensions are described in this definition: the quality of the overall training process and the quality of specific training sessions. In this context, multiple training sessions together form the overall training process. Here, quality of the process refers to how well it prepares the athletes for performing at the desired level. This may involve factors like structured planning, training load management, and regular evaluation, while quality of training sessions requires deliberate physical, technical, and mental execution to develop the skills needed for competition [1, 2]. These dimensions are interconnected, with each session acting as a building block within the broader training process. It is common practice to periodize the training process in different cycles, where sessions are parts of a macrocycle (year(s)), mesocycle (month(s)), and micro-cycle (week(s)), following Matveyev’s classic model [3].

In the context of both the training process and specific sessions, the coach–athlete cooperation is central to supporting athletes’ performance development and well-being [4]. Through coaches’ professional, pedagogical, and interpersonal skills, they help structure daily training and guide athletes toward achieving competitive goals [1, 4]. Côté and Gilbert refer to coaching effectiveness as the application of integrated professional, interpersonal, and intrapersonal knowledge that may improve athletes’ competence, connection, confidence, and character. Coaches act as discussion partners, organizers, and supervisors, offering feedback and guidance to address technical, mental, or physiological needs [2, 5]. This dynamic may foster a strong coach–athlete relationship, which is suggested as key to promoting athlete development [6, 7]. The coach–athlete relationship is seen as mutually dependent and central to coaching effectiveness [6, 8], with trust, autonomy support, and sport-specific knowledge being particularly important [5, 9].

Coaches of para-athletes are expected to be personally supportive and able to adapt to athletes’ prerequisites [10, 11]. Para-athletes have identified the coach as a key contributor to enhanced training quality through individualized coaching, open dialogue, and interdisciplinary collaboration [12]. The athletes’ training is influenced by impairment-related factors [13], which may introduce additional complexity in achieving high training quality [14]. A large body of research has highlighted that para-athletes is a very heterogenous group without a one-size-fits all approach [15, 16]. Previous research has highlighted several barriers for sport participation amongst para-athletes related to health [17–19], sports facilities [20, 21], and logistics [12, 22] which coaches should consider in the planning, execution and evaluation of training. Different impairment characteristics may require unique coaching- and training strategies for achieving high training quality. Thus, coaches of para-athletes play a key role in adapting both the training process and individual sessions to the athlete’s specific prerequisites [12, 23]. Therefore, a coach’s ability to understand each para-athlete’s unique condition stands out as essential.

While limited research has been conducted on training quality, one study explored which factors elite para-athletes across different sports and impairments associated with training quality [12]. From a process perspective, coaches’ role was important for personal and individualized coaching to enhance training quality, while from a session perspective, coaches ensured daily dialogue and calibration of athlete’s condition. In addition to the coach and support team, important factors influencing training quality for para-athletes included goal setting, load management, technical execution, and solution-oriented modification of training [12]. These are factors that coaches normally manage through close cooperation with the athlete [24], and their ability to reflect and learn from practice has been suggested as a success criterion for developing effective coaching strategies [25].

Coaching in para-sport may require a deeper knowledge and understanding of impairments, health considerations, and the need for inclusive environments [26, 27]. Elite para-sport coaches’ experiences can offer valuable guidance for others working in Paralympic sport that aim to increase their athletes’ training quality. In a recent commentary [1], Bucher Sandbakk and coworkers raise several important questions, among them: “Which factors influence training quality?” Proposed key factors associated with training quality include athletes’ optimization of session- preparation, execution and evaluation. Overall, the coach-athlete interplay is emphasized as important for the quality of both the training process and specific sessions. In this context, coaches having extensive sport-specific knowledge, experience and pedagogic skills are suggested to enhance training quality, through good training plans, continuous communication and session adjustment. However, these are mainly general considerations and do not focus specifically on para-athletes.

While para-athletes’ perspectives on training quality have been explored [12], no study has investigated what factors coaches of para-athletes perceive as important for high training quality in para-sport. Given their central role in both planning, execution, and evaluation, we consider coaches to be uniquely positioned to share their experiences related to training quality. By focusing on coaches who have successfully guided athletes to Paralympic and World Championship levels, we aim to identify transferable insights that can inform coaching practice across para-sport contexts. Accordingly, we aimed to explore what factors elite para-sport coaches perceive as important for achieving high training quality.

## Methods

### Research design and epistemology

We conducted a qualitative study using a hermeneutic phenomenological approach to explore what coaches working with elite para-athletes consider important for training quality. This approach is situated within the constructivist paradigm and aligns with ontological relativism and a subjectivist epistemology, and it assumes that reality may be experienced differently by each coach [28, 29]. As suggested by Guba and Lincoln [30]: “we construct meaning based on interactions with our surroundings”. In this context, the surroundings may include various coaching situations as well as the complexities of coaching elite para-athletes, implying heterogeneity in aspects such as impairment characteristics, performance demands, and specific equipment [13]. Moreover, understanding the factors influencing training quality is based on comprehensive dialogue and interpretation, a process that can be influenced by, for example, the researcher’s pre-understanding. Therefore, a hermeneutic phenomenological approach was chosen because its epistemology assumes that knowledge is co-constructed and acknowledges the researcher’s interpretive role [31]. Thus, to obtain methodological coherence [32], semi-structured interviews was chosen to gain rich, in-depth insight into coaches’ lived experiences (the parts) and interpret what they viewed as necessary to achieve training quality (the whole) [31].

The interview guide was structured around the two dimensions of training quality proposed by Bucher Sandbakk and co-workers [1]: (a) training quality in specific sessions and (b) training quality in the overall training process. These dimensions emerged from their interpretations of best practice literature and from personal dialogues with world-leading athletes and coaches across different sports [1]. Bucher Sandbakk and co-workers further argue that training quality depends on the context, making it challenging to reach full consensus on the concept. Accordingly, to fully explore coaches’ unique perspectives on training quality, we adopted a hermeneutic phenomenological approach.

Regarding the research team’s positionality, the main author (VØ) has a background in human movement science, including research and practical coaching in endurance sports and para sports. The co-authors also have backgrounds combining sports research and practice, spanning cross-country skiing, cycling, orienteering, soccer, and strength training. Every member of the team has experience with para-related research; however, aside from the first (VØ) and last (ØS) authors, the others have limited experience in coaching elite para-athletes. None of the authors have a disability.

Data was analyzed inductively using reflexive thematic analysis [33] to iteratively identify and interpret meaning across coaches’ experiences and construct common themes. Given the researchers’ active role in this process, we applied a hermeneutic phenomenological lens throughout data collection and analysis. This approach allowed for a dynamic interpretation of coaches’ perspectives in relation to their specific training contexts and the characteristics of their athletes [31].

### Study participants

Coaches were recruited via professional networks and direct email invitations. All participants provided written informed consent prior to participation. Eligible participants were current or former coaches of Norwegian elite para-athletes at international level. To ensure a broad range of elite-level experience, we included coaches from different sports, working with athletes with different types of impairment. A minimum of three years of experience from coaching para-athletes was required to ensure that participants had a substantial coaching background to draw from.

Eight current or former elite para-sport coaches, representing eight different para-sports, participated in this study (see Table 1). By the eighth interview, recurring ideas indicated that we had reached thematic saturation. For confidentiality, each coach is referred to by an anonymized ID (Coach 1–8) when quoting their remarks.

**Table 1:** Description of the coaches participating, and the impairments and sports of their athletes

Demographics	
Number of coaches (n)	8
Age (mean, ± sd)	46.5 ± 9.5
Years of coaching experience* (mean ± sd)	22.1 ± 11.6
Years of para-sport coaching experience (mean ± sd)	11.8 ± 9.6
Impairments (n)	5
Cerebral Palsy, Limb deficiency**, Muscle deficiency, Spinal cord injury***, Visual impairment	
Sports represented (n)	8
Alpine skiing, Badminton, Climbing, Cross-country skiing, Cycling, Rowing, Swimming, Table-tennis	

Data are presented as mean ± sd for continuous variables and n

* Including experience coaching in para-sport

**Including amputation and congenital limb deficiency

*** Including spina bifida

### Ethical considerations

The research project was approved by the Norwegian Agency for Shared Services in Education and Research (SIKT, reference no. 441007) prior to data collection. All participants were informed about the study’s purpose, participation guidelines, data storage, research team members, publication plans, and their right to withdraw at any time. To contextualize their experience, we present the type of sport the coaches worked in; however, to prevent identification, we do not report coaches’ gender or link specific sports and impairments to individual quotes. Each coach reviewed the manuscript before submission to ensure they agreed with the content.

### Data generation

Semi-structured interviews were conducted between October 16, 2023, and October 17, 2024, typically at locations familiar to the coaches. Each interview began with a brief explanation of the project’s purpose and the researcher’s background, followed by introductory questions about the coach’s background and experience in building rapport and context.

The interview guide (Supplementary file) was divided into two parts: (a) training quality in a specific session and (b) training quality in the overall training process. A 10-minute break separated the two parts. Part (a) and Part (b) had average durations of 62 and 44 min, respectively, with a mean total interview time of 106 min. Coaches were encouraged to provide examples when answering, for example when relating their experiences to athletes with different impairments. Interviewers allowed ample time for elaboration, and follow-up questions and prompts were used to explore relevant topics in depth. Although the interview guide separated session-specific and process-specific questions, we remained attentive to information that transcended this division. Any misunderstandings were clarified to ensure mutual understanding. Seven out of eight interviews were conducted in Norwegian, while the eighth in English. All interviews were audio-recorded with participant permission and transcribed verbatim for analysis.

### Analysis

We followed the six-step reflexive thematic analysis approach of Braun and Clarke [34] to explore the coaches’ lived experiences of what they perceive important for training quality in both session and process contexts. The first author (VØ) led the analysis and engaged in regular dialogue with co-authors to identify, structure, interpret, and construct overarching themes and subthemes that represented shared meanings across the data.

#### Familiarization

The first author immersed in the data by listening to all interview recordings while reading the transcripts to ensure accuracy and gain a holistic understanding.

#### Coding

All transcripts were analyzed inductively. We coded semantically for surface meanings and latently for deeper interpretations where needed. Here, the first author’s prior experience helped in interpreting what the coaches’ experiences implied. For example, when discussing how athletes could work on improvements to enhance the quality of their sessions, one para-cycling coach stated: “*That is an important step for athletes for them to develop that internal sense of how they feel. Then they need to practice it. And this is a really good way for them to practice evaluating themselves and their sense of the training. How did that feel? Did I control my training? Well*,* how tired am I?*” To identify the deeper meaning, this was latently coded as “Self-reflection and evaluation after sessions can strengthen the athletes’ awareness related to the session goal.” Initially, we coded session-related and process-related content separately, yielding 266 codes for session-related content and 186 codes for process-related content.

#### Generating initial themes

Codes reflecting similar experiences were grouped into initial themes, resulting in 30 session-related themes and 25 process-related themes.

#### Reviewing themes

We examined and refined these initial themes. Initially, we organized them into two overarching themes (each with subthemes) for each interview part. After reviewing the thematic structure with co-authors and reflecting on researcher subjectivity, we observed substantial overlaps between the session and process categories. We therefore merged the structures into four overarching themes, that were mutual for session and process, each encompassing two subthemes.

#### Defining and naming themes

We further refined the overarching themes and subthemes, differentiating their relevance to session versus process quality using filled versus unfilled symbols in our data display (see Table 2). In this way, the first author (VØ) drew on his background to interpret and understand differences in the relevance of quality factors between the overall training process and specific sessions. Co-authors critically assessed the clarity and distinctiveness of each theme and subtheme, and also interpreted the relevance of each subtheme. They applied their prior knowledge in sports research to ensure that the themes were understandable to potential end users.

#### Writing up

The authors reached consensus on the final overarching themes and subthemes. The quotes and themes were translated from Norwegian into English, with ongoing discussions to ensure clear expression and accurate meaning. The results were written with illustrative quotes to represent the coaches’ experiences in a coherent narrative.

### Methodological integrity

To manage the risk of bias (confirmability), we practiced reflexivity throughout the research process. The lead researcher’s prior experience coaching elite para-athletes [35] was continuously reflected upon when developing codes and themes, to ensure that it contributed to deeper understanding rather than creating biased perspectives. Co-authors posed critical questions about interpretations and ensured that themes were grounded in the coaches’ own accounts, enhancing credibility and transparency [30, 36]. Reflexivity regarding how the researchers’ subjectivity might influence the analysis was an ongoing part of our discussions.

We adopted a hermeneutic phenomenological approach, which is situated within the constructivist paradigm and has a subjectivist epistemology that acknowledges the researcher as an instrument in the research process [28, 29]. As such, it recognizes that the researcher’s prior experience can both enrich and bias the interpretation of qualitative data [30]. To address this, we practiced reflexivity throughout the research process. The main author’s background in coaching elite para-athletes [35] was explicitly acknowledged and regularly discussed within the research team. This background facilitated a deeper interpretation of the coaches’ experiences; for example, in understanding how an impairment influences daily training, how coach–athlete cooperation during sessions is managed, and how adaptations in training facilities may influence the athletes before and during sessions. Co-authors critically examined interpretations, posed challenging questions, and reviewed coding and theme development to ensure that the findings were grounded in the participants’ accounts rather than the researchers’ preconceptions. This collaborative process enhanced the credibility and transparency of the analysis [30, 36] .

To further strengthen methodological rigor, we adhered to the Consolidated Criteria for Reporting Qualitative Research (COREQ) guidelines, which promote transparency and comprehensive reporting in qualitative studies. Each coach was given the opportunity to review and comment on the manuscript prior to submission, which served as a form of participant validation and increased the trustworthiness of the findings.

## Results

Through reflexive thematic analysis, the experiences of elite para-sport coaches revealed four overarching themes that they perceived as important for achieving high training quality among their athletes: [1] a supportive training environment [2], organization and adaptation of the training process [3], coaches’ understanding of the athlete and sport, and [4] athletes’ ownership of training. Notably, during the semi-structured interviews, coaches described largely the same factors as influencing training quality both in the overall training process and individual training sessions, rather than entirely distinct factors for each. After discussions conducted to refine the themes, Table 2 presents these factors (overarching themes and their subthemes), indicating their relative relevance to the training process and to specific training sessions.

**Table 2:**
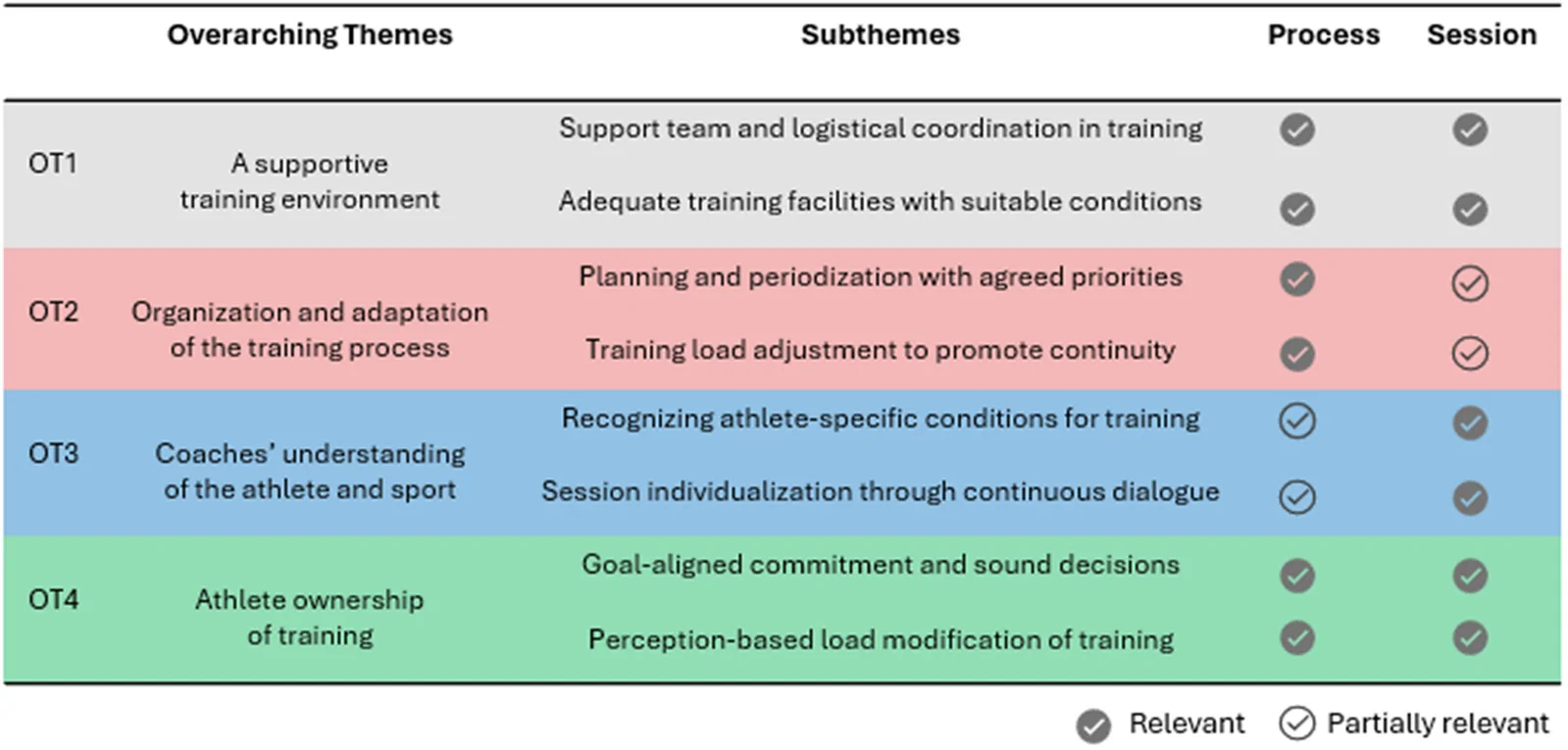
Identified overarching themes (OT) and subthemes showing what coaches perceive as important for elite para-athletes to achieve high training quality. Filled symbols denote the subtheme is relevant, and unfilled symbols denote partially relevant, to the training process or to specific training sessions

**Fig. 1:**
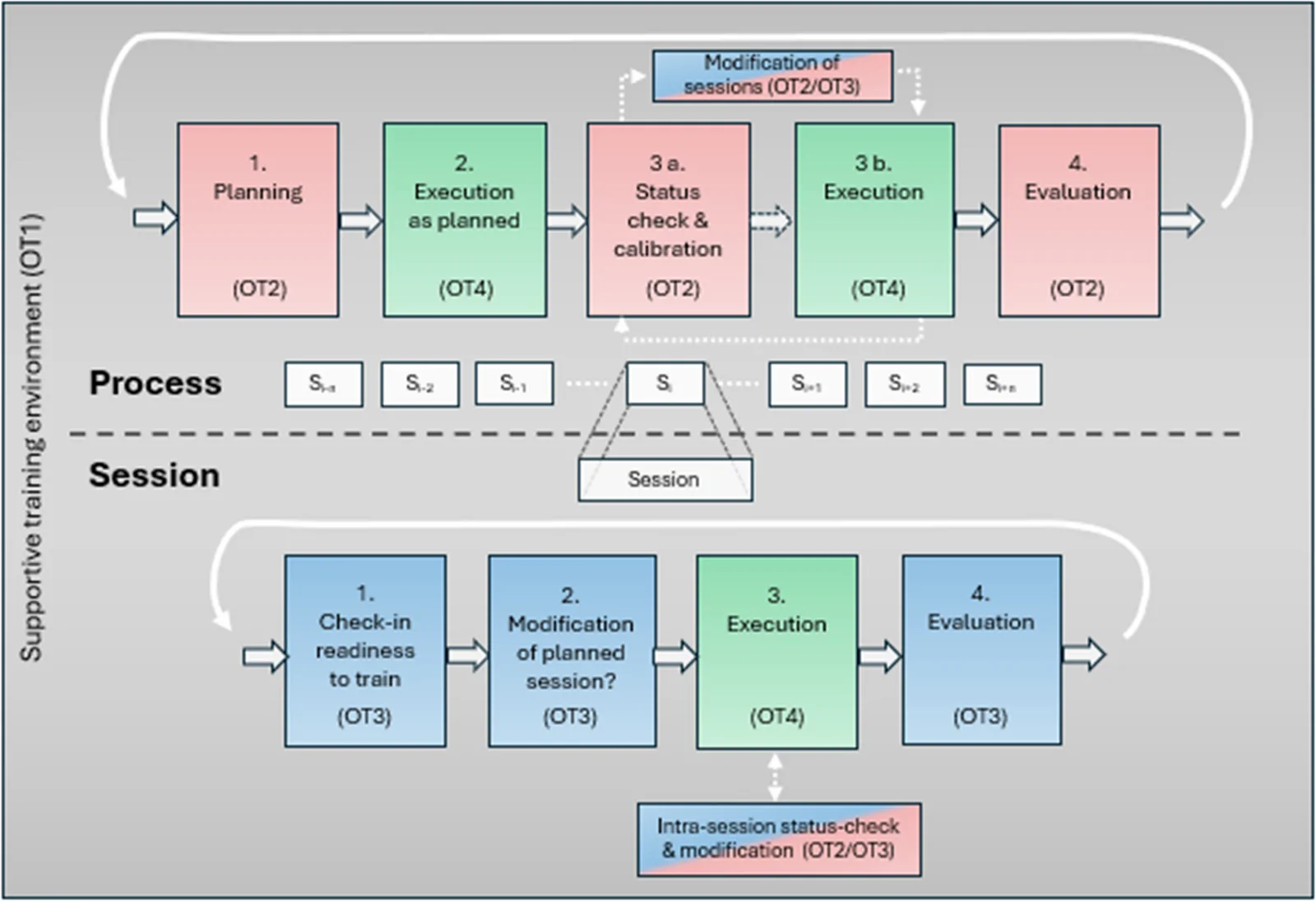
Workflow for achieving high training quality, illustrating the coach–athlete collaboration across the training process (top) and training sessions (bottom). Colors indicate which overarching themes (OT1–OT4) are most relevant at each stage. The workflow operates within a supportive training environment (OT1). OT2 is the organization and adaptation of the training process, OT3 is coaches’ understanding of the athlete and sport, and OT4 is the athlete ownership of training. For process, training progresses through: (1) planning training blocks with agreed priorities, (2) execution as planned, (3a) status monitoring and calibration, (3b) modified execution based on athlete conditions, and (4) evaluation. Multiple sessions (S₁.Sₙ) make up the overall process. At the session level, each session includes: (1) a check-in to assess athlete readiness, (2) possible modification of the planned session based on readiness, (3) execution of the session, and (4) evaluation. An additional intra-session feedback loop allows ongoing status checks and adjustments during execution

The four overarching themes emerged as interwoven in a *collaborative workflow* involving the coach, the athlete and the support team, to achieve high training quality. This collaboration spans planning, execution, and evaluation of both the overall training process and individual sessions. Figure 1 illustrates this workflow for training quality, highlighting how the coach–athlete collaboration operates before, during, and after training sessions and across the training process.

### OT1) A supportive training environment

#### Support team and logistical coordination in training

The analysis revealed that well-integrated support teams were regarded as highly important for training quality by the coaches, which included people covering different roles and responsibilities. They helped achieving high training quality by filling expertise gaps and by supervising training, although these positions were covered to different extents within the teams. Many coaches underscored the importance of physical presence during training sessions, creating a training environment that enabled immediate feedback, pre- and post-training evaluation, and fine-tuning of session elements, that could ensure training quality. One of the coaches exemplified what roles the support team could include:

Coach 4: *“I think the support team is essential [for achieving training quality] in para-sport. Besides me as a coach*,* the support-team may include for example physiotherapists*,* personal assistants*,* trainers from the national elite sport center*,* assistant coach*,* healthcare personnel*,* parents*,* or orthopedic engineers”.*

Regarding the overall training process, many coaches emphasized that the level of financial support within a sport federation affected whether and where they could hold training camps, the extent to which coaches could be present in daily training, support staff assistance and the level of equipment customization. During training camps, the coach and support team cooperated organizing transportation, ensuring training facilities (and related amenities such as restrooms, lodging, meals) were accessible, and managing equipment. This facilitated training quality by removing administrative stress before, during and after sessions for the athletes. As one coach explained:

Coach 1: *“For the training process*,* I think the best athletes have the ability to put in consistent training and deliver day by day*,* week by week. […] That also involves that they have a structure around them where they can afford to train daily*,* either through a stipend or some other sort of funding. And that they’re in an environment where they can train daily. Without others or a support team around them*,* you just can’t*,* very few people can do it totally on their own”.*

#### Adequate training facilities with suitable conditions

Our interpretation of what coaches experienced as a foundational need to achieve high quality training, focused on daily access to training facilities with suitable conditions. From a process perspective, many highlighted that athletes’ daily commute to suitable training facilities was critical for sustaining quality training over time. Difficult transportation for wheelchair users during winter or long travel times for athletes with cerebral palsy or visual impairment were said to drain time and energy, negatively impacting athletes’ ability to execute quality sessions. Some coaches gave examples of training camps where training quality often improved due to the proximity of lodging, training venues, and dining, which minimized transportation required. From a session perspective, coaches stressed that facilities must be adapted to be accessible for athletes with different impairments. Several noted the importance of having certain areas within facilities reliably available and prepared for their para-athletes’ use. One coach highlighted how they tried to gain information, but needed to be solution oriented:

Coach 7: *“Sometimes we [the coach and support team] must plan sessions on site depending on what we have available and what we are able to organize. For our sport*,* we are particularly concerned about adapted facilities so that logistics work smoothly. Considering wheelchairs*,* it is important that the facilities are accessible so that the athletes can access them on their own wherever possible. […] So*,* we are dependent on trying to find as much information as possible before going places”.*

Training alongside teammates in a well-equipped facility created suitable conditions for enhancing both training quality and athlete well-being. Many coaches observed that having training partners at similar level improved the competition-relevance. In some sports, a low number of para-athletes was reported as challenging for the session quality. In this regard, some coaches arranged sessions where para-athletes trained together with and against non-disabled athletes, while others focused on creating a strong group of similar-level para-athletes in sports where this was important. One coach addressed the challenges for athletes that did not train among others:

Coach 1: *“For para-athletes*,* one of the huge difficulties is there are just very few of them out there. So*,* they don’t get a chance to train with other para-athletes regularly*,* often not until they’re at a competition. […] Because when you are in a race*,* you need to be strategic and tactical*,* so we need to do that [to achieve training quality]. We put a big effort into our training camps*,* because that’s where we bring everyone together and work with other athletes”.*

### OT2) Organization and adaptation of the training process

#### Planning and periodization with agreed priorities

All the coaches elaborated on how thorough planning was the basis for a high-quality training process. Many coaches emphasized collaboration with the athletes when developing training plans and defining goals. Incorporating athletes’ view on periodization, expected training load, competition schedules, and planned camps enhanced their commitment to the plans and further the quality of the process. Many coaches emphasized their discussions with their athletes as valuable, examining what qualities to develop in certain periods and how specific sessions fit into the long-term process. Having the athletes understand and believe in the process plan was seen as essential for conducting several quality sessions over time. In this context, minor day-to-day adjustments could be made to achieve high training quality, without deviating from the direction of the process. One coach pointed out how planning could help maintain direction:

Coach 7: *“Clear goals and good planning done in advance are important to avoid making things up as you go and help to ensure a clear thread through the process. If necessary*,* it is okay to make minor daily adjustments*,* while still maintaining a clear direction. I highlight planning as a key factor for [training quality] success because it helps you better handle unexpected events”.*

Structured planning was reported by all the coaches to facilitate daily training predictability. An agreed training schedule made it easier for the athletes to achieve high session quality, due to adequate preparation of equipment, school, work or family management, and logistical coordination with guides, assistant coaches or training peers. In a process perspective, coaches noted that a structured plan made it easier to monitor and calibrate athlete progression through testing, competition results, and evaluations. Based on results, plans could be further modified with the athlete to maintain both process and session quality while ensuring progress toward defined goals. One coach exemplified this as following:

Coach 8: *“We structure the training process in 4-week blocks because it is relatively short and clear. […] We touch on technical*,* tactical*,* physical and mental components*,* which I believe makes it easier to track progress. […] It is easier for the athlete to plan and prepare if they know what we will focus on for the coming weeks. […] Ahead of each block*,* we agree on what we want to develop and why*,* which makes it easier to structure the blocks for competitions"*.

#### Training load adjustment to promote continuity

The analysis repeatedly showed that the coaches emphasized continuity in training as a key factor for ensuring high quality of a training process. In this regard, the adjustment of total load served as crucial for long-term development. Importantly for para-athletes, the coaches emphasized that impairment-related issues, such as fatigue, daily use of wheelchair or crutches, as well as secondary health conditions had a considerable effect on the total load. Balancing these factors was perceived challenging; coaches were concerned with injury prevention, sufficient recovery, and minimizing energy “losses” due to logistics or social factors. Some coaches observed that the athletes with the best training quality were skilled at managing these factors, suggesting that successful adjustment of training load is a common ingredient in maintaining quality training. One coach stated:

Coach 6: *“The total load for an athlete is the sum of the load from activities of daily living and the load from training […] One should keep in mind that the total load influences the level of training quality. [As a coach] You know that some para-athletes have somewhat different prerequisites for carrying out their sessions*,* which is sometimes easy to forget for both the athlete and coach. Communication and awareness are therefore important”*.

We interpreted the coaches’ experiences as reflecting the athletes having an intuitive self-awareness that helped them maintain continuity in training. Coaches gave examples of athletes having a “gut feeling” for when to back off or push harder, to balance several sessions in a row. Such adjustments helped athletes obtain consistency in training, achieving process quality by adhering to a certain session standard. This awareness typically increased with years of experience and athletic maturity. Coaches noted that as their athletes gained experience, deeper discussions occurred that could further improve training quality and performance. One coach explained what training consistency encompassed:

Coach 1: *“I think consistency is extremely important for quality. When we talk about process*,* we’re talking about doing multiple sessions a week*,* week after week. It’s not about ‘smashing it’ one day and then being totally dead for the next two days. That’s not good quality training. It’s more about consistency. And that means going into each training*,* remembering what you did yesterday and thinking about what you’re going to do tomorrow”.*

### OT3) Coaches’ understanding of the athlete and sport

#### Recognizing athlete-specific conditions for training

Through the analysis, it emerged that all coaches adopted an external perspective on their coaching role where they reflected on what they considered as valuable contributions to their athletes’ training quality. Coaches agreed that they could improve their athletes’ training quality through continually developing competence to understand each athlete’s individual prerequisites. This includes understanding how impairment relates to performance demands, movement capabilities, and medical issues, such as pressure sores, challenges in the cold, or fatigue. This type of knowledge also helped the coaches adapt the training sessions to fit athletes with different characteristics. To improve the quality of the process, several coaches stated they had to understand the consequences of the impairment from a wider perspective. This forced them to think one step beyond standard sport specific knowledge when considering i.e. load regulation, equipment modifications, facility adaptations, and travel logistics. One coach underlined the importance of drawing attention to specific aspects related to the athletes’ impairments:

Coach 3: *“What is important for para-athletes’ training quality is that we must educate more coaches who have knowledge of para-sport*,* adaptation and creativity in training. […] We have come a long way with things being somewhat equal [between para and non-disabled athletes]*,* but equal is not good enough for para-athletes. We must prioritize what is truly best for para-athletes*,* which I feel is sometimes overlooked. Some believe that doing what non-disabled athletes do yields the best results*,* but in reality*,* it doesn’t”.*

#### Session individualization through continuous dialogue

It became apparent through the analysis that communication was closely linked to training quality. All coaches reported that individualizing sessions was based on a frequent dialogue with their athletes. Many coaches felt that improvements in training quality and performance were linked to improvements in the coach–athlete relationship. Honesty, trust, and openness fostered a culture where thoughts and feedback could be shared freely. They described how they worked closely with each athlete to identify specific elements to improve and how to customize different sessions. This helped the athletes to mitigate barriers to training quality and focus on their possibilities rather than limitations. In practice, coaches ensured frequent check-ins before, during, and after sessions. This gave them a thorough understanding of the factors affecting each athlete on a day-to-day basis. The following extraction highlights how this allowed them to individualize sessions in real time:

Coach 6: *“[…] You must know enough about the athlete to understand how to communicate*,* what type of communication to use in different situations*,* and to read their body language. […] I have established three routines for each session: we have a check-in*,* a mid-session check*,* and a check-out. That has contributed to increased sharpness for both me and the athletes to lift the training quality*,* because they know there is an expectation when the session begins: a clarification at the start*,* a calibration during*,* and a short evaluation afterward”.*

When reflecting about the training quality of different athletes, the coaches expressed similar views regarding the benefit of paying attention to athletes’ personal day-to-day experiences living with an impairment. By asking open-ended questions, listening, and communicating well, coaches could learn more about their athletes’ backgrounds, how they experienced different tasks in a session, and what the coach could do to improve those tasks. All coaches viewed the athletes as experts on their own bodies, meaning the athletes knew best how a movement or exercise should feel when done correctly. Therefore, session plans could be customized or even changed ‘on the fly’ based on athletes’ perceptions. Coaches aimed for deliberate individualization to optimize training quality, although they noted that some athletes initially resisted having their training altered. This could challenge training quality at times but was usually resolved through open dialogue and a trustful relationship. One coach illustrated the flexibility needed for achieving high training quality:

Coach 7: *“Dialogue and discussion during the session are important when coaching para-athletes. We may have planned an exercise in advance and thought it would work*,* but then we tried it with the athlete and it’s a totally different story. They don’t feel it where we expected them to*,* and the whole exercise must be scrapped. So having that dialogue during the session is hugely important to make the sessions as good as possible”.*

### OT4) Athletes’ ownership of training

#### Goal-aligned commitment and sound decisions

When reflecting on both the quality of the training process and specific sessions during the interviews, the coaches consistently highlighted the importance of athletes’ ownership of training. The athlete’s understanding of each session’s purpose and how multiple sessions are interconnected, was considered important for training quality. Coaches described the importance of athletes making informed decisions to keep each session aligned with its intended purpose and overall goal. This was illustrated by the most successful athletes as often self-reflecting before and during sessions and engaging in dialogue with the coach to deliberately assess their readiness and execution. This helped them gauge their physical capacity for high quality execution and make good decisions accordingly. One coach included this as part of the coaching philosophy:

Coach 3: *“As a coach*,* I aim to support the athletes in becoming their own coach. […] Independent of which of us that have planned the session*,* we are discussing the content before we start. I have an inquiry-based philosophy*,* so I ask questions about their thoughts*,* to enhance their awareness. Our dialogue continues during and after the session*,* considering how they experience various tasks”.*

The most successful athletes were experienced to accept the day-to-day variations in physical and mental readiness and adjust their preparation and execution according to these and the session goals. At the same time, these athletes considered the entire week or training cycle when modifying session or process plans, making sure that any changes still fit within the larger plan. Ultimately, coaches referred to successful athletes’ persistent commitment, which led them to diligently complete even “boring but essential” training—thus enhancing the quality of the overall process. One coach said:

Coach 5: *”Athlete readiness is important for training quality. We know why we are conducting the session*,* according to a clear session goal. Regarding para-athletes*,* one must give greater conisderaton to day-to-day readiness variations. The athlete has worked on the preparations*,* which sometimes begins 24 hours ahead.”*

#### Perception-based load modification of training

Athletes’ perception of intensity and load during sessions was considered by many coaches to be an important tool for achieving high training quality. Some coaches noted that how well athletes adjusted intensity according to day-to-day variations in body condition, weather, temperature, and opponents, was often determined by the athlete’s level of perception and regulation of effort. Coaches underscored that only the athlete truly knows how certain intensities and movements feel; therefore, they maintained a close dialogue with the athletes, that created awareness during training execution. In contrast, athletes who failed to achieve high training quality were described as those who only focused on “finishing” the session, rather than self-reflecting and working deliberately on each task. One coach demonstrated the self-reflecting process for an athlete trying to achieve high training quality:

Coach 1: *“I think step one for an athlete is: OK*,* am I reaching my targets [power and heart rate]? […] Step two is understanding how those two behave on some days. […] Understanding that difference so that they can*,* on a daily level*,* customize*,* adapt*,* and modify their training based on their feeling. […] The third is the feeling itself. That sense includes the feeling of the blood in your legs*,* the lactic acid*,* the burn in your lungs*,* the feeling of your pulse*,* your feeling of your heart rate and your breathing rate. […] I think all three of those coming together is what shows quality training”.*

The coaches noted that assessing and adjusting macro-, meso- and microcycles, was a continuous collaboration between the athlete, coach and support-team, but underscored that it was based on athlete initiatives. They expected athletes to be aware of their energy levels and attending bodily signals, to be accordingly proactive in modifying upcoming sessions. In this context, they spoke about the importance of athlete ownership to fine-tune the execution of sessions, highlighting the athlete’s responsibility to regularly share their perceptions through the process and during session preparation, execution, and debrief. For example, one coach reflected on the differences between athletes’ level of experience:

Coach 2: *“A plan is made to be changed. […] The final touch is the individual adaptations made along the way [by the athlete]*,* based on experience and gut feeling. There are differences between athletes: less experienced feel they need to follow the plan to consider training as high quality*,* whereas the experienced athletes think that adjusting sessions actually improves training quality”.*

## Discussion

In this study, we aimed to explore which factors elite para-sport coaches perceive as important for achieving high training quality, to identify transferable insights that can inform coaching practice across para-sport contexts. Through a reflexive thematic analysis, applying a hermeneutic phenomenological approach, the coaches’ experiences revealed four overarching themes associated with training quality both relating to the training process and specific training sessions. In essence, achieving high training quality involves a supportive training environment (OT1), the organization and adaptation of the training process (OT2), the coach’s understanding of the athlete and sport (OT3), and the athlete’s ownership of training (OT4) (Table 2). All these factors are dependent on a well-established, close and continuous collaboration between the coach, the athlete and the support team (Fig. 1). Another take away from this study is to include an intra-session cycle, in which within-session adjustments are done to optimize training quality, thereby adding a fourth level to Matveyev’s periodization model [3].

### Adequacy of the training environment

The importance of adequate training environment involved adapted training facilities that could facilitate high-quality training among para-athletes with different impairments. This indicates that a training facility needs to be universally designed for different impairments and allow para-athletes to execute sport-specific activities in the facility. Elite para-athletes have previously noted factors such as custom equipment configurations, facility lighting, and suitable conditions indoors and outdoors, as beneficial for training quality [12]. Other studies report that poorly adapted training facilities can be a challenge for e.g. wheelchair users, or visually impaired athletes, causing poor training conditions [21, 37, 38]. Well-adapted facilities can help coaches and athletes focus on session objectives rather than logistical issues, and through enhanced session efficacy and task completion, improve training quality. While non-disabled athletes likely take these aspects for granted, the importance of having access to suitable facilities may be larger for para-athletes and make a greater impact on training quality. We understood this theme as reflecting that coaches and support teams of para-athletes will likely benefit from considering the facilities level of adequacy when aiming for high quality in training processes, sessions and camps.

### The significance of support teams

Support teams were highly valued, ensuring training quality by filling competence gaps and assisting with practical tasks. This suggests that a support team covering important roles, tailored to the athletes’ specific needs, influence training quality positively. In previous research, a well-integrated support team has been found to offer competence that may enhance athlete well-being [39, 40], discuss and refine performance factors [24, 41], and likely facilitate training quality. Our results align with elite para-athletes’ impressions reported by Øvstehage and coworkers [12], that described how their support-team contributed to high-quality training through assistance and supervision in strength and endurance training. In light of the large heterogeneity in athlete characteristics in para-sport described by Webborn and Van de Vliet [13], coaches and athletes may consider seeking people with specific expertise when considering challenges related to physiotherapy, physiological testing, medical practice, or sport and impairment-specific equipment. The two latter may be especially important regarding para-athletes’ training quality and performance [42, 43]. Hence, coaches and athletes may ensure improved training quality by organizing a support team that is based on inter-disciplinary collaboration covering the needs of every athlete in the team.

### Frequent dialogue to manage training load

The coach-athlete calibration dialogue, comparing status with pre-set plans, is perceived to be necessary for adjusting load and maintaining high training quality (Fig. 1). This finding emphasizes that coaches need to ensure calibration routines with frequent dialogue to capture both major and minor changes in the athletes’ life. Sandbakk and coworkers [24] reported that elite endurance coaches have previously stressed athlete involvement, and careful load–recovery management to achieve high-quality sessions. From a health perspective, elite para-athletes have a notable incident of illness and injury, often linked to their impairments [19, 42–45]. Moreover, external stressors outside sport (e.g. logistics and activities of daily living), have also been identified as challenging among elite para-athletes [12, 46, 47]. The close coach-athlete dialogue including session check-ins stands as a promising method to gauge daily conditions. Establishing such regular monitoring can help coaches catch warning signs and avoid overtraining [14, 42, 48], where such iterative learning processes, may help to individualize and modify training, further improving process and session quality [1, 4, 25] (Fig. 1). In practice, periodic coach–athlete debriefs to recalibrate the process can yield adjustments such as modification of current plans, equipment or goals, that improve quality in subsequent sessions or training weeks. Adequate adjustment of load may thus not only optimize performance and reduce illness and injury rates but consequently enhance training quality.

### Coaches’ understanding of impairment implications

Our results indicate that coaches should gain knowledge to better understand the combination of an athlete's impairment and sport to help them achieve training quality. Accordingly, coaches need to be curious and open-minded in their role, to constantly improve their ability to grasp the unique characteristics of every individual so they can optimally fit their training philosophy to the needs of each unique athlete. This has also been reported by elite para-athletes, that noted the importance of having coaches that can understand the mechanisms related to their impairment [12, 48].

On the other hand, Cregan and coworkers [5] found coaches in para-sport to argue that para-athletes should be coached as athletes first, rather than focusing on their disability. Accordingly, Allan and coworkers [7] reported para-athletes to emphasize that coaches should respect the athlete’s expertise about their own body. While there may be differing opinions about the extent to which and how coaches should consider the impairment, elite para-athletes highlighted the importance of a coach that possessed sufficient knowledge to understand the relation between impairment, sport, and training quality [12]. Accordingly, and according to our coaches, the impairment should not become the sole focus, but the coach must grasp its influence on training quality. It appears that coaches are required to find the balance between having knowledge necessary to understand the implications of being a para-athlete, but at the same time recognize the athletes’ expertise on their impairment.

### Athlete autonomy

Coaches perceived training quality to be achieved through collaborative decision-making; however, they also highlighted athletes’ autonomy and their ability to make sound decisions as key. This means that the athlete must develop their ability to be the director of their own training project, and at the same time work closely with their coach, to achieve high training quality. Banack and coworkers [9] suggested that fostering a safe and supportive training environment, may empower athletes to make deliberate choices, likely facilitating athlete ownership of training. In this context, elite coaches have highlighted autonomy-supportive factors such as athlete commitment, post-session reflection, and coach–athlete discussions as a basis for learning to improve training quality [1, 24].

Following Øvstehage and coworkers’ [12] exploration of key factors influencing training quality, taking ownership may imply deliberate choices regarding athlete-controlled factors such as preparation, energy distribution, self-awareness, and modification of training. Given the various factors that may influence elite para-athletes’ life [12, 42, 46, 49, 50], optimization of quality itself may rely on the athletes’ ability to tailor their choices to their current life-, impairment- and training conditions. In such cases, athletes make initial decisions, while relying on the coach as a reflection partner and educator that can help optimize training quality [10, 25]. Coaches should be aware of the fine-tuned balance between fostering athlete independence and providing coach supervision. Thus, providing enough space for the athlete to self-reflect and assess different strategies seems advantageous. Our interpretation was that this was a part of athletes’ ownership and responsibility of initiating collaborative decisions towards higher training quality.

### Collaborative decision-making

Taking together these overarching themes, they emphasize the importance of close and ongoing collaboration between the coach, the athlete and the support team to achieve training quality. It seems that the calibration, dialogue and modification of training is driven by athlete’s ownership of training, serving as a catalyst for the initiatives that ensure training quality both in the training process and specific sessions (Fig. 1). Given that many Paralympic sports operate with relatively limited resources [21, 22, 46], athlete proactive behavior seems crucial for taking advantage of a team collaboration that can optimize quality indicators [12]. The coach–athlete collaboration has been highlighted by Canadian para-athletes as one of the main factors underpinning athlete development in para-sport [7]. They describe this as a dynamic process of co-creating knowledge, something our coaches also affirmed, emphasizing continuous interaction with the athlete as key to achieving training quality. Despite having different roles, the coach’s and athlete’s contributions to training quality appeared mutually dependent in our study; each one’s efforts enabled the other’s perspective to be fully realized. Thus, we interpret this as indicating that a dynamic and multi-disciplinary collaboration across the process and session dimensions facilitates discussion that can lead to high training quality.

### Adding a fourth level of Matveyev’s periodization model

Matveyev’s classic periodization model presents three main cycles; macro-, meso- and micro-cycles. Within these cycles, training sessions are aligned to produce the best possible adaptations and aim for a performance peak at a specific time. Within training sessions, coaches and athletes cooperate to optimize the various components of session execution (for example, by modulating intensity, making small tweaks to strength or technical exercises, or inserting extra recovery as needed) to achieve high training quality. If a session begins too hard or an athlete is having a bad day, immediate modifications are required, and these will inevitably influence the wider training process in the days that follow. Our findings support adding a fourth intra-session cycle to encompass the adjustments and fine-tuning occurring within each training session (OT3) (Fig. 1). This intra-session cycle can be viewed as the lowest level of the periodization hierarchy. Changes made at this level can affect the microcycle and mesocycle, and vice versa, reinforcing the interconnection between the training process and sessions as suggested by Bucher Sandbakk et al. [1]. In practice, the coach and the athlete engage in a continuous feedback loop, learning and adjusting both within each session and between sessions, to optimize training quality.

### Limitations and future directions

We acknowledge several limitations. First, the study was conducted in Norway, and experiences may differ in other national or cultural contexts. Second, the authors of this study do not have lived experience of having a disability, which makes it more challenging to interpret and understand the subjective impairment-specific aspects of the data material. We can see how the impairment influence athletes, but we might not fully grasp the consequences of being disabled [51]. Third, to protect participant confidentiality, we did not link specific sports or impairments to individual quotes, which may slightly reduce contextual richness and transferability. However, the inclusion of coaches from eight different sports and five impairment categories suggests that the insights are relevant across a range of para-sport contexts. Practitioners should adapt these findings to the specific needs and circumstances of their athletes.

Notably, our findings correspond with many factors that para-athletes themselves have identified as important for training quality [12], indicating shared perceptions between athletes and coaches about what matters most. As our study centered on elite para-sport coaches, future research should explore perceptions of training quality among elite coaches of non-disabled athletes, as well as coaches and athletes within para-sport at the development level. Such work could reveal which factors are universal and which are unique to the para-sport context and at different levels, further informing best practices for coaching across different athlete populations.

### Practical applications

Based on the findings of this study, we recommend the following practical measures of high training quality for coaches and support staff working with elite para-athletes. Implementing these measures can help improve training quality and, ultimately, support better performance development and athlete well-being among elite para-athletes.

#### Ensure adapted and accessible training environments

Provide facilities, equipment, and logistical arrangements that accommodate athletes’ impairment-specific needs, thereby removing barriers to high-quality training.

#### Plan collaboratively and flexibly

Establish a structured training plan with clear long-term goals in partnership with the athlete but retain flexibility for day-to-day adjustments based on the athlete’s condition and feedback.

#### Develop impairment-specific knowledge and understanding

Engage in understanding the athlete’s impairment and how it affects their training and performance, enabling more effective adaptations and informed decision-making.

#### Foster continuous dialogue through structured routines

Maintain regular check-ins and open communication before, during, and after sessions. Use these conversations to monitor the athlete’s status and collaboratively adjust training in real time within the intra-session cycle.

#### Empower athlete ownership

Involve athletes in training decisions and encourage them to reflect on their training process. By supporting their autonomy and self-awareness, they increase their commitment and accountability for training quality.

#### Leverage the support team

Utilize assistant coaches, medical and sport science staff, guides, and even experienced peer athletes to fill knowledge gaps and assist with training tasks. A well-integrated support team can provide comprehensive support and expertise, enhancing the overall training environment.

## Conclusions

Elite para-coaches perceive training quality to be dependent on a dynamic collaboration between the coach, the athlete, and the support team. Our findings suggest that high training quality is achieved through close dialogue and understanding between the coach and athlete, supported by the athlete’s ownership of their training within a supportive environment. This indicates that training quality must be optimized before, during, and after training, through preparation, calibration, and evaluation respectively, within both the overall training process and individual sessions. Altogether, this study informs para-coaching practice by suggesting perceived factors that may improve training quality from both a process perspective and a session perspective.

## Supplementary Information

Supplementary Material 1.

## Data Availability

The datasets generated and/or analyzed during the current study are not publicly available due to risk of identification through the combination of statements, sports and athletes’ impairments, but are available from the corresponding author on reasonable request and with permission of the participants.
