# Supplementary material for: Coaches’ perspectives on training quality in elite para-sport: a qualitative study of coach–athlete collaboration
Source: BMC Sports Sci Med Rehabil. 2026 Jun 10;18:383. doi: 10.1186/s13102-026-01788-5 (PMC13520284; doi:10.1186/s13102-026-01788-5)
Supplement: Supplementary file 1 [file 13102_2026_1788_MOESM1_ESM.pdf]

<u>**Interview guide Training Quality, Coaches in para-sport**</u>

To begin with, we need some information about you (coach):

Age?

Sex?

Sport?

For how long have you worked as a coach in sports?

For how long have you worked as a coach in para-sport?

For how long have you worked as a coach in the para-sport you are coaching at the moment?

For how long have you worked as a coach for the national team in the para-sport you are coaching at the moment?

Why are you a coach in the para-sport you are coaching at the moment?

<u>**Introduction**</u>

In this interview we will focus on training quality.

What do the expression «training quality» imply? OR What do you put into the expression «training quality»?

What characterizes good training quality?

In part 1 of the interview, we are going to talk about what factors that characterize and influence training quality in specific training sessions.

In part 2 of the interview, we are going to talk about what factors that characterize and influence training quality in the holistic training process.

SESSION

In this interview we are going to talk about how you as a coach in para-sport consider the training quality of your para-athletes. You are coaching several para-athletes with different impairments. (Examples may be athletes with visual impairment, congenital limb deficiency or spinal cord injury, that in turn provides the athletes with different prerequisites in their daily training). It is important that you have your athletes and their prerequisites in mind when you answer, and also try to relate your answers to specific impairments.

**1**	*Think of some really good sessions that your athletes have executed. What factors were important for attaining good training quality in those sessions?*
1.1	*Are these factors, that are important for good training quality, dependent of what kind of session it is? For example: Low intensity vs. High intensity sessions?*
1.2	*You mentioned these factors _________ , but what do you think about factors important for the quality of physical/mental/technical aspects?*
1.3	*You mentioned the execution of a session, but what do you think about factors in the preparation for a session?*
1.4	*Are there any prerequisites that need to be present for your athletes in order to attain good training quality in the session?*
1.5	*How do the athletes secure that factors that are important for good training quality, really become good?*
1.6	*Think of some really bad sessions that your athletes have executed. What factors were important for attaining bad training quality in those sessions? (OR ..important for bad training quality to occur in those sessions?)*
**2**	*How do your athletes assess or measure training quality for a specific session?*
2.1	*Do the athletes assess their training quality in every session or is there a difference dependent of what kind of session it is?*
2.2	*Based on your definition of training quality ___________: If the athletes need to adjust or modify the session compared to the plan, how will you then describe the training quality of the session?*
2.3	*How often does it happen that the athletes need to adjust or modify the session?*
2.4	*What is usually the reason when athletes need to adjust or modify the session?*

**3**	*If there are any, what barriers or obstacles for attaining good training quality in specific sessions have you identified among your athletes?*
(3.1)	*Which of these barriers or obstacles are related to the athlete's impairment?*
3.2	*What are typical factors that create these barriers/obstacles?*
3.3	*In what way do you experience these barriers to influence the quality of the session for the athletes?*
3.4	*Do the athletes have any strategies to reduce or manage these barriers or obstacles?*
3.5	*You have mentioned barriers related to ___________, but what do you think about aspects like for example the environmental, the logistical, the physical or the mental aspect?*
3.6	*The ability to manage these barriers, is that what distinguishes the best para-athletes and the others?*
3.7	*Are there any impairments that have better prerequisites for attaining good training quality in a specific session than others?*
3.8	*What factors important for good training quality are there the biggest differences in between the best para-athletes compared to the other athletes?*

Repeat question 1 and ask if there is anything that the coach wants to add after part 1.

PROCESS

We will now talk about training quality in a training process for your para-athletes. A training process is regarded as a longer period consisting of several sessions that is organized to give certain adjustments over time, and the related tools and processes that are applicated to reach the best possible result. A training process may include everything from daily choices to long term planning, execution, and evaluation. It is important that you still have your athletes and their impairments in mind when we now are going to talk about quality in a training process.

**1**	*Think of a really good training process that your athletes have executed. What factors were important for attaining good training quality in that process?*
1.1	*You mentioned factors in _________ phase. Are there any other factors you see as important for the athletes' quality in the training process?*
1.2	*Are there any prerequisites that need to be present to attain good quality in the training process?*
1.3	*How do the athletes secure that factors that are important for good quality in the training process, really become good?*
1.4	*Think of a really bad training process that your athletes have executed. What factors were important for attaining bad quality in that training process? (OR ..important for bad quality to occur in that training process?)*
**2**	*How do your athletes assess or measure the quality of the training process?*
2.1	*How often do the athletes assess the quality of the training process?*
2.2	*If it happens, what can be the reasons when athletes need to adjust or modify the execution of a training process?*
**3**	*If there are any, what barriers or obstacles for attaining good quality in a training process have you identified among your athletes?*
(3.1)	*Which of these barriers or obstacles are related to the athlete's impairment?*
3.2	*What are typical factors that create these barriers/obstacles?*
3.3	*In what way do you experience these barriers to influence the quality of the session for the athletes?*
3.4	*Do the athletes have any strategies to reduce or manage these barriers or obstacles?*

3.5	*The ability to manage these barriers, is that what distinguishes the best para-athletes and the others?*
3.6	*Are there any impairments that have better prerequisites for attaining good quality in a training process than others?*
3.7	*What factors important for good quality in the training process are there the biggest differences in between the best para-athletes compared to the other athletes?*

Repeat question 1 and ask if there is anything that the coach wants to add after part 2.

Notes:

____

____

____

____

____

____

____

____

____

____

____

____

____

____

____

____

____

____

____

____

____

____
